# Nutritional and exercise interventions to improve conception in women suffering from obesity and distinct nosological entities

**DOI:** 10.3389/fendo.2024.1426542

**Published:** 2024-06-28

**Authors:** Evdoxia Gitsi, Sarantis Livadas, Georgia Argyrakopoulou

**Affiliations:** ^1^ Diabetes and Obesity Unit, Athens Medical Center, Athens, Greece; ^2^ Endocrine Unit, Athens Medical Center, Athens, Greece

**Keywords:** fertility, obesity, nutrition, exercise, PCOS, lifestyle intervation, type 2 diabetes (T2D), hypothyroidism

## Abstract

Infertility among women, particularly those living with obesity, presents a multifaceted challenge with implications for reproductive health worldwide. Lifestyle interventions, mainly focusing on weight loss, have emerged as promising strategies to improve fertility outcomes in this population. This review aims to explore the effectiveness of various lifestyle interventions, encompassing dietary modifications and exercise regimens, in enhancing fertility outcomes among women with obesity and associated conditions such as polycystic ovary syndrome, congenital adrenal hyperplasia, type 2 diabetes mellitus, premenopause, hypothyroidism and eating disorders. Methodology of study search encompass a broad spectrum, ranging from interventions targeting weight management through slow or rapid weight loss to dietary approaches emphasizing whole food groups, specific nutrients, and dietary patterns like low-carbohydrate or ketogenic diets, as well as the Mediterranean diet. By synthesizing existing findings and recommendations, this review contributes to the understanding of lifestyle interventions in addressing infertility, with an emphasis on the population of women of reproductive age with excess weight and known or unknown infertility issues, while promoting their integration into clinical practice to optimize reproductive health and overall well-being.

## 1 Introduction

Infertility is a widespread health concern, affecting a significant portion (20–30% globally) of women in reproductive age (1). Defined as the inability to achieve clinical pregnancy after 12 months of regular unprotected sexual intercourse, infertility has multifaceted causes ranging from functional to anatomical abnormalities within the reproductive system (1–3). Among these causes, ovulatory dysfunction, particularly prevalent in conditions such as polycystic ovary syndrome (PCOS), stands out as a leading cause of female infertility (2).

Compounded by the global rise in obesity rates among women of reproductive age, the intricate interplay between obesity and infertility has gained attention. Obesity, characterized by an excessive accumulation of body fat, poses a substantial risk factor for infertility, adversely affecting various aspects of reproductive health (4). By 2025, it is projected that approximately 20% of women of reproductive age will be affected by obesity (5, 6). Given this fact, excess weight among women planning to conceive could potentially hinder successful conception (4). In particular, studies have shown that women with obesity are at significantly higher risk of ovulatory-disorder infertility and are prone to experiencing complications such as miscarriages and poor pregnancy outcomes (7, 8). Potential mechanisms of the obesity-infertility linkage are disruptions to the hypothalamus–pituitary-ovarian (HPO) axis, mediated by hormonal imbalances and metabolic perturbations associated with excess adiposity (3). Hyperandrogenism, insulin resistance (IR), and altered mitochondrial activity emerge as pivotal factors influencing folliculogenesis and oocyte quality (3, 9).

Given these insights, addressing obesity through lifestyle modifications has garnered attention as a promising strategy for enhancing fertility outcomes, especially in women with excess weight. To support this viewpoint, it is essential to recognize that lifestyle factors, including reduced physical activity and unhealthy dietary habits, contribute significantly to the increasing incidence of infertility (7, 10, 11). Moreover, lifestyle interventions, such as weight loss and lifestyle modification programs offer a cost-effective approach at a national level (12). Therefore, it is imperative to delve deeper into evaluating their efficacy and implementing suitable interventions in clinical practice.

This review aims to explore diet and exercise interventions and strategies, aimed at directly or indirectly improving fertility outcomes in women living with overweight and obesity, along with associated conditions such as PCOS, congenital adrenal hyperplasia (CAH), type 2 diabetes mellitus (T2DM), premenopause, hypothyroidism and eating disorders (EDs).

## 2 The impact of lifestyle modification

In the context of lifestyle interventions to enhance fertility in women dealing with overweight or obesity with no other evident medical causes of infertility, considerable focus has been directed towards the effectiveness of preconception intensive lifestyle approaches within the scientific literature. Notably, most of the studies focus on promoting weight loss and have demonstrated improvements in fertility among women with obesity, mainly increasing the likelihood of spontaneous pregnancies.

A systematic review and meta-analysis examining the impact of preconception lifestyle interventions on fertility highlighted their positive influence on weight loss and increased natural pregnancy rates. However, the interventions did not exhibit significant effects on live birth or birth weight outcomes (13). Likewise, a second meta-analysis emphasized that reduced-calorie diets and exercise interventions in women were more likely to result in pregnancy (risk ratio 1.59, 95% CI 1.01 to 2.50), primarily through the mechanisms of weight loss and improved ovulation. Importantly, these modifications did not show a reduction in miscarriage rates (14), while on the contrary Espinós et al., observed a potential elevation of the later through weight loss interventions, despite the slight elevation of pregnancy rates (15).

A randomized controlled trial (RCT) involving women aged 18–38 with obesity, intending to become pregnant, implemented a more restrictive dietetic program comparing a very low energy diet (VLED) in the intervention group with a standard dietary intervention (SDI) in the control group. The VLED group exhibited substantially higher weight loss (11.9%) compared to the SDI group (3.1%) at the end of the 12-week intervention. Moreover, women in the VLED group experienced a significantly shorter time to pregnancy, particularly evident within 90 days of the intervention (16). Integrating dietary changes, including a 6-week VLED followed by SDI, exercise, and behavioral components, with assisted reproductive technology (ART) treatment in women with obesity resulted in the intervention group achieving a higher pregnancy rate (48% vs. 14%; P = 0.007), requiring fewer fertility treatment cycles per pregnancy (mean two vs. four in the control group; P = 0.002), and witnessing a substantial increase in the number of live births (44% vs. 14%; P = 0.02) (17). Expert guidance aligns with these findings, recommending that women with obesity and infertility should prioritize adopting a healthy lifestyle, emphasizing weight loss, before undergoing either natural conception or medically assisted reproductive techniques (18).

While most data indicate that weight loss interventions are an effective way to promote ovulation and thus pregnancy, certain studies fail to establish significant impacts of lifestyle modifications on direct measures of fertility. Specifically, a preconception intensive lifestyle intervention for weight loss in women with obesity and infertility, despite improvements in metabolic health, did not demonstrate enhanced fertility or birth outcomes when compared to an exercise intervention without a focus on weight loss (19). Targeting the same population, a lifestyle intervention preceding infertility treatment did not result in higher rates of healthy singleton vaginal births at term within 24 months after randomization, despite achieving modest weight loss (mean reduction of 4.4 kg vs. 1.1 kg in placebo) (20). Importantly, the rates of pregnancy-related, labor-related, and neonatal complications, as well as neonatal outcomes, remained comparable between the intervention and control groups (20). A recent meta-analysis involving 27 randomized controlled trials (RCTs) with a mean cohort body mass index (BMI) of 30 kg/m^2^ or above suggested that weight loss interventions did not significantly increase pregnancies compared to minimal interventions or controls. However, the interpretation of this outcome should be approached cautiously due to limited fertility-related data across the included trials (21). Regarding the isolated impact of physical activity on fertility outcomes in women with obesity, a sub-analysis within a large cohort study indicated that high levels of physical activity did not mitigate the heightened infertility risk in women with obesity. This suggests that a high BMI plays a pivotal role in this relationship, and the protective effects of physical activity are primarily observable in women with a normal BMI (22).

Concerning the specific components of lifestyle intervention programs, it is recommended that a combination of reduced calorie intake by moderating fat and reducing refined carbohydrate intake, along with increased aerobic exercise, should be the cornerstone for individuals facing obesity and infertility (14, 23). Studies on women of reproductive age, irrespective of weight status, indicate that diets high in trans fats, refined carbohydrates, and added sugars can adversely impact fertility (24). In a large prospective study, each 2% increase in energy intake from trans unsaturated fats compared to that from carbohydrates, was associated with a 73% higher risk of ovulatory infertility. Similarly, with the former compared to monounsaturated fats, more than a doubled risk of ovulatory infertility was observed, even after adjusting for known risk factors (25).

Despite these findings, no specific macronutrient composition appears superior for improving reproductive outcomes in women of reproductive age, as very-low-carbohydrate diets, in contrast to low-fat diets, showed no statistically significant effect on pregnancy outcomes (21). However, an observational study in female mice found that exposure to a high-fat diet (HFD) led to a significant decrease in primordial follicles, impacting fertility. This effect was observed independently of obesity, with the latter being a deteriorating factor, and was associated with higher proinflammatory cytokine levels and increased ovarian macrophage infiltration. It is advisable to maintain moderate fat intake until further research clarifies the exact molecular mechanisms behind these outcomes (26).

Protein intake should align with recommendations for the general population, considering factors such as physical activity levels, and it is advisable to prioritize plant protein sources (24). The prospective Nurses Health Cohort Study II (NHS II) indicated that substituting 5% of energy from animal protein with plant protein could decrease anovulatory infertility risk by more than 50%. This outcome may stem from the varying impacts of plant and animal proteins on insulin and insulin-like growth-factor I (IGF-I) secretion, with plant protein’s potential fertility benefits likely arising from its weaker insulin response compared to animal protein (7).

Moreover, while moderate caffeine and alcohol consumption appears neutral in its impact on fertility, high caffeine intake may prolong the time to achieve pregnancy and increase the risk of pregnancy loss. The recommended dose for pregnant women or those attempting pregnancy is up to 200 mg of caffeine per day. Notably, heavy drinking and chronic alcohol consumption are linked to reduced fertility and a higher risk of menstrual disorders (24).

Regarding specific dietary patterns, adherence to a Mediterranean-type diet, rich in dietary fiber, omega-3 fatty acids, plant-based protein, vitamins, and minerals, demonstrates a positive impact on female fertility (24). High adherence to a Mediterranean-type pattern has been associated both with improvement of natural and clinical pregnancy, i.e. before *In vitro* fertilization (IVF), as well as live birth among women under 35 years old (27, 28). Certain individual substances found in the Mediterranean diet (MD), including fibers, polyphenols, vitamins C and A, β-carotene, and folate, indirectly promote reproductive health by reducing oxidative stress, enhancing insulin sensitivity, and modulating immune system responses via beneficial effects on the gut microbiome (29). However, the collective benefits of the MD are likely due to synergistic effects rather than isolated components. MD impacts reproductive health through complex mechanisms involving gene expression, epigenetic modifications, and signaling pathways (30). Additionally, the reproductive benefits of the MD may intersect with other lifestyle factors; for instance, individuals with high adherence to the MD often exhibit other healthy behaviors such as regular physical activity, non-smoking habits, and lower stress levels (29). As previously highlighted, it is important to proceed with caution when extrapolating these findings to populations with a BMI exceeding 25 kg/m², given the focus on individuals with normal weight in these studies. Moreover, in a cross-sectional study, the pro-fertility diet, characterized by whole grains, soy and seafood, low pesticide residue produce, supplemental folic acid, B12, and vitamin D, has been linked with significantly improved markers of ovarian reserve, including a higher serum anti-mullerian hormone (AMH) concentration and antral follicle count (AFC), indirectly contributing to enhanced fertility (31).

**Figure 1:**
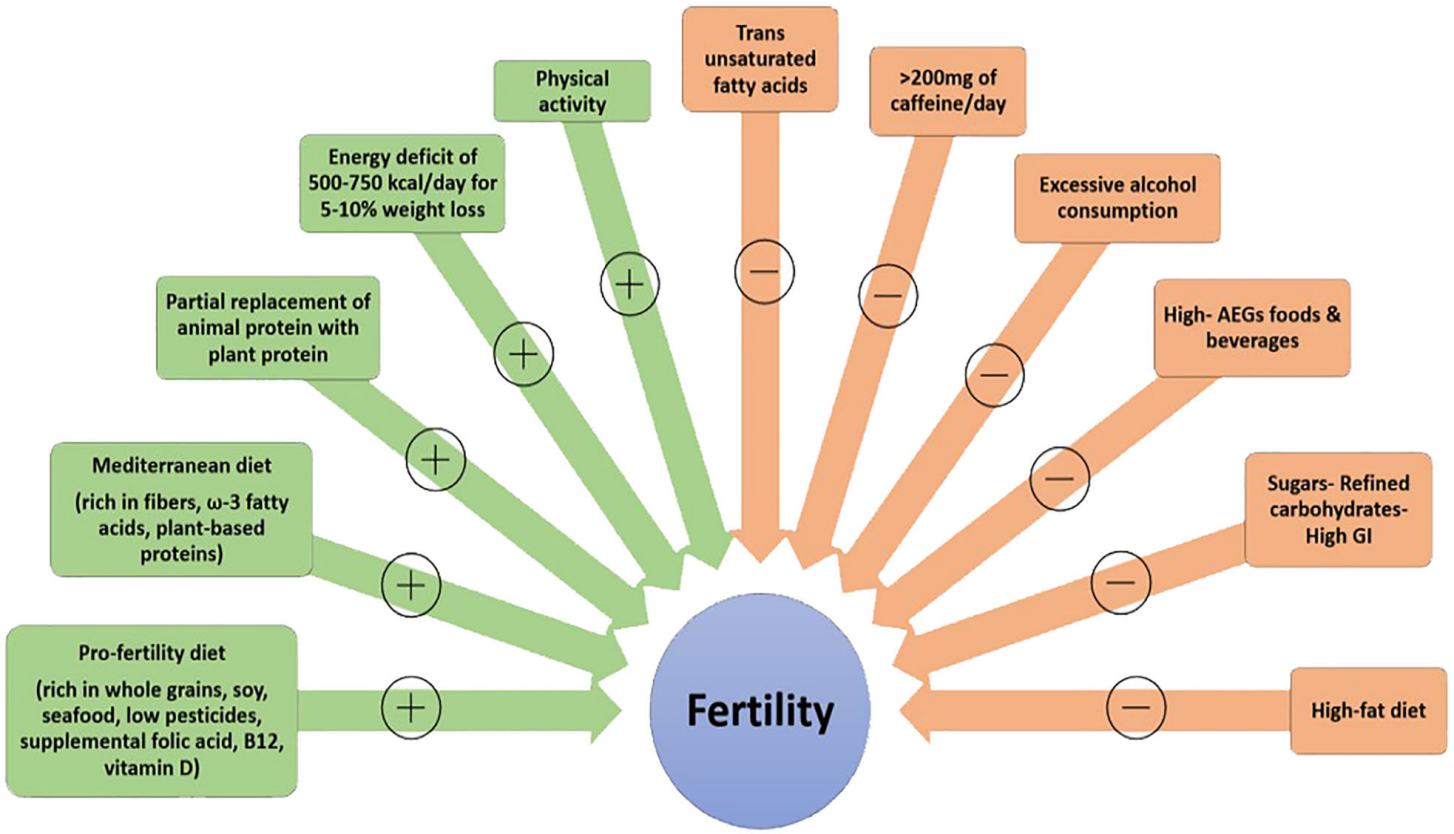
Lifestyle factors potentially affecting fertility (+: positively, -: negatively) AGEs, advanced glycation end products; GI, glycemic index.

Overall, adopting a diet emphasizing nutrients associated with positive fertility outcomes such as dietary fiber, omega-3 fatty acids, and plant-based proteins, while avoiding refined carbohydrates, trans fatty acids, overconsumption of coffee and alcohol, which are linked to negative impacts, alongside regular exercise and maintenance of a normal weight, represents an effective lifestyle approach to enhancing reproductive health and fertility (see **Figure 1**).

## 3 Women with overweight/obesity and PCOS

PCOS is a prevalent endocrine and metabolic disorder affecting approximately 7–20% of women in reproductive age (32, 33). PCOS, characterized by symptoms such as androgen excess (manifesting as hirsutism and/or hyperandrogenemia) and ovarian dysfunction (presented as oligo-ovulation and/or polycystic ovarian morphology) (34), poses an increased risk of infertility (35). One of the primary underlying mechanisms regulating syndrome’s pathophysiology is IR. In fact, IR constitutes an inherent disorder in women with either normal or high BMI and PCOS exacerbating every aspect of the syndrome. Regarding fertility there is a well-known direct link of IR with chronic anovulation as well as increased miscarriage and pregnancy loss (36). It is obvious that obesity further deteriorates subfertility, by increasing the degree of IR by almost 15% (37). It has been estimated that 75% of women dealing with overweight or obesity and infertility issues have PCOS, with the former posing additional challenges to fertility due to higher levels of visceral adiposity, disturbances in IR, sex steroid metabolism and regularity of menstrual cycles (38–41).

Several therapeutic approaches have been implemented the last decades for subfertility in PCOS, and despite drug treatment (e.g. metformin, letrozole, inositol) (42), lifestyle adjustments, such as modifications in nutrition and exercise, are deemed the fundamental approach for these patients, particularly in the context of overweight and obesity (32). Notably, weight loss may be more advantageous for women with PCOS compared to those without PCOS, as they often exhibit increased central obesity and IR for a given level of obesity (18). The existing evidence points towards nutrition and exercise interventions ability to demonstrate a normalization of ovulation function and menstrual cycles (38, 43), thereby increasing the likelihood of successful pregnancies in women with PCOS with overweight and obesity (44–47). In particular, a study involving women with obesity, PCOS and infertility issues in a six-month lifestyle intervention, in addition to metformin plus clomiphene medication, demonstrated significant improvements in reproductive endocrine profiles, lipid metabolism, and a reduction in the volume of both left and right ovaries. In contrast to medical treatment alone, the intervention led to increased rates of menstrual recovery, ovulation, and pregnancy (47). Interestingly, when lifestyle modifications precede potential medical treatment with clomiphene citrate and/or metformin, in addition to IR reduction, the aforementioned improvements in the menstrual cycles, ovulation and pregnancy rates appear to be similar or even more robust, compared to immediate treatment (45, 48). The efficacy of lifestyle modifications is highlighted by Oberg et al., who demonstrate enhancements in reproductive function after a 4-month behavioral modification intervention, incorporating current knowledge on weight control, personal leadership, mindfulness, and information on physical activity and diet, along with both group and personal support. Despite a modest weight loss of minus 2% in the intervention group (p<0.05), there was a notable improvement in menstrual regularity for 25% more women in the intervention group, compared with minimal intervention. Additionally, 38% of women aspiring to conceive successfully achieved pregnancy within one year of completing the study (46).

While it is advisable to lower calorie intake and promote weight loss in women with PCOS and excess weight, existing dietary recommendations for PCOS often rely on studies involving women with obesity without PCOS (49). Studies indicate that a modest weight loss of 5% to 10%, achieved through moderate caloric restriction over 6 months, can enhance metabolic and reproductive variables in women with obesity and infertility (32, 49). To this direction, guidelines for PCOS assessment and management suggest creating an energy deficit of 30% or 500–750 kcal/day (with an energy provision of approximately 1200–1500 kcal/day), tailored to individual parameters, such as energy requirements, body weight, and physical activity levels (49). Most studies focusing on direct reproductive outcomes found no significant differences between various dietary patterns and there is still no consensus regarding preferable nutritional treatment for women with PCOS and increased BMI (49, 50). Fundamentally, an hypocaloric diet is proved clinically beneficial, irrespective of its composition (49, 50).

However, current data on diet and female infertility suggest that specific dietary modifications could benefit women with PCOS by preventing low-grade chronic inflammation and improving mainly indirect reproductive outcomes (51). A low to moderate carbohydrate and low-glycemic index (LGI) diet is frequently recommended for women with PCOS (52). Reduced carbohydrate intake has been linked to reduced IR and an improved hormonal profile in women with PCOS and overweight or obesity (53, 54). This, in turn, may enhance ovulatory function and reduce ovulatory infertility (53, 54). A systematic review, including studies with carbohydrate intake below 45%, highlighted significant improvements in menstrual cyclicity and ovulation rates with low-carbohydrate diets, indicating potential fertility optimization in this population (55). There is some evidence corelating ketogenic diets (usually providing <30–50g of carbohydrates) with superior effectiveness in terms of more rapid weight loss, improved IR, and promotion of ovulation compared to conventional hypocaloric diets (56, 57). Additionally, ketogenic diets have been shown to elevate sex hormone binding globulin (SHBG), decrease circulating levels of free testosterone, and reduce the luteinizing hormone to follicle stimulating hormone ratio (LH/FSH) (56). Interestingly, when a Very Low- Calorie Ketogenic Diet (VLCKD) (500–800 kcal, with high-biological-value protein) was administered to reproductive-aged women with PCOS and obesity in a RCT, it proved superior to a mediterranean low calorie diet (1200–1400 kcal) in reducing BMI, fat mass, waist circumference, fasting insulin, HOMA-IR, total cholesterol, and increasing SHBG, leading to decreased free testosterone. Additionally, the VLCKD significantly improved ovulation, with a 46.1% increase compared to 21.4% in the control group, potentially benefiting fertility (58) This finding, coupled with others associating VLCKD with maintained energy expenditure, enhanced physical activity, and reduced food cravings, implies that, under proper patient selection and medical supervision, due to the complex biochemical implications of ketosis and the strict therapeutic compliance required, VLCKD could be effectively and safely employed to induce rapid weight loss (57). Moreover, higher protein intake may be more effective at suppressing androgen levels in women with PCOS compared to high carbohydrate diets (52). In a RCT involving women with PCOS and obesity, a modified hypocaloric diet with high protein and low glycemic load, compared to a conventional hypocaloric diet, led to reductions in insulin, HOMA-IR (a marker of IR), and androgens, with an increase in sex hormone-binding globulin (SHBG), in the absence of insulin-sensitizing agents usage (59). Additionally, LGI diets showed promise in improving menstrual regularity in up to 80% of women following them (50, 60).

Concerning fats, HFDs may negatively impact ovarian follicles, potentially impairing fertility (61). Trans fatty acids (TFAs) are associated with an increased risk of ovulatory infertility in women with PCOS (62). Considering the evidence, long-term low carbohydrate diets, possibly in combination with a moderate fat composition, may be recommended for BMI reduction, improved IR and low-density lipoprotein cholesterol (LDL-C) levels, increased FSH and SHBG, and decreased testosterone levels (55).

In addition to adjusting macronutrient composition, reducing consumption of high- advanced glycation end products (AGEs)-(a varied group of highly oxidizing compounds with significant pathogenic roles in diabetes and other chronic diseases)-foods and adopting a diet with lower cooking temperatures and specific methods like boiling, poaching, and steaming can improve metabolic and hormonal profiles while reducing oxidative stress in women with PCOS (63, 64). The aforementioned intervention included instructing participants to limit red meat, poultry, and fish to once a week, opting for boiled or steamed foods such as pasta, vegetables, and legumes, and eliminating high-AGEs fast food and beverages. This confirms existing evidence linking Western dietary patterns with adverse effects on endocrine metabolism and potentially fertility (24). In fact Diamanti-Kandarakis group has provided significant evidence that AGEs are actively involved in the pathogenesis of PCOS development through inflammatory and oxidative stress pathways. Moreover, reduction of AGE’s dietary consumption was directly related to improvement of IR and hyperandrogenemia, in women with PCOS, showing on clinical grounds the importance of directed nutritional intervention and the effect on woman’s physiology (64).

In adjunction to dietary considerations, individuals with PCOS should follow general exercise guidelines, despite limited studies directly assessing the impact of exercise on ovulation or pregnancy rates in this population (45, 49, 60, 65). An intervention involving a pedometer and at-home exercise with weekly step-count goals showed positive outcomes in weight loss and fertility, with 40% of weight loss participants achieving pregnancy (66). Although significance was not reached, potential reasons include the small sample size and the low to moderate intensity of aerobic exercise. Contrary to concerns in healthy reproductive-aged women, vigorous exercise appears most beneficial for women with PCOS, even correlating with improved chances of conception with as little as 30 minutes of vigorous exercise three times per week (60). Notably, the fertility benefits of exercise in women with PCOS extend beyond weight reduction, as demonstrated in a large prospective study by Rich-Edwards et al., where the association between vigorous exercise and reduced risk of ovulatory infertility persisted after adjusting for BMI (67–69).

For effective management of subfertility in PCOS, tailored lifestyle interventions emphasizing nutrition modifications, including low-carbohydrate, LGI diets, alongside high-protein, moderate to low-fat compositions, coupled with regular moderate to vigorous exercise, are recommended to facilitate weight loss and improve metabolic and hormonal profiles, ultimately enhancing ovulation and reproductive outcomes.

## 4 Women with overweight/obesity and CAH

CAH encompasses monogenic autosomal recessive disorders, primarily due to 21-hydroxylase deficiency, leading to classical and non-classical forms, namely patients with total or partial cortisol production (70, 71). Diagnosis relies on clinical symptoms and usually a PCOS like syndrome is usually observed in women suffering from the non-classical form of the disorder (72). Excess adrenal androgens, chronic anovulation, and endometrial dysfunction contribute to reduced delivery rates in females with 21-hydroxylase deficiency (73–75). In fact higher rates of spontaneous miscarriage have been reported. However, hydrocortisone administration in supplementation dose (10–20mg daily) and not at pharmacological range is usually sufficient to reduce androgen excess and improve ovarian function and ovulation (75). Genotype analysis in the mother and in some cases in the prospective father is highly recommended in order to avoid the birth of children suffering from the classic form of the disease where glucocorticoid supplementation is mandatory throughout life (75).Lifestyle interventions, such as optimizing dietary habits, incorporating regular exercise, and considering weight loss when indicated targeting the aforementioned factors, as applied to women without CAH, could be recommended for managing weight gain, preventing or treating obesity, and addressing possible IR, hypertension, and dyslipidemia in patients with CAH, potentially contributing indirectly to improved fertility outcomes (76–78). Additionally, adequate protein, calcium, and vitamin D intake are essential for bone health, with protein intake recommendations possibly aligning with populations at risk of muscle loss (1–1.5g/kg/day), although it is not scientifically specified for this group of patients (77). Vitamin D supplementation may be necessary in regions with low fish consumption or inadequate sunlight exposure (77). While adhering to sodium intake recommendations of around 1500mg per day is generally advised, patients with salt-wasting classic CAH may necessitate a higher salt intake, and, therefore, strict salt restriction is not advised in this case (76). Considering the heterogeneity of CAH patients undergoing glucocorticoid therapy, recommendations should be regarded as general guidelines adaptable to individual needs, preferences, and goals (76).

## 5 Women with overweight/obesity prior to menopause

A woman’s ability to conceive i.e. fertility, diminishes gradually with age. With individual variations, this decline starts around 30 years old, intensifies between 35 and 40, and dramatically accelerates thereafter. Reproductive menopause marks the inability to achieve pregnancy, while actual menopause typically occurs after a significant decline in conception potential by about 10 years (79). ARTs refer to fertility treatments involving the manipulation of eggs or embryos to assist individuals experiencing difficulty in achieving pregnancy naturally (80). However, women with overweight and obesity undergoing ART experience reduced success rates, compared to those with a BMI <25 kg/m^2^, attributed to diminished ovarian response, suboptimal oocyte quality, and adverse endometrial changes (81). Consequently, it is essential to address the significance of maintaining a healthy weight with patients, emphasizing effective interventions that integrate nutritional education and exercise counseling (82). In this case, achieving a 5%–10% reduction in total body weight over a 6-month period, accompanied by lifestyle modifications, has demonstrated efficacy in mitigating the severity of obesity-related risk factors, enhancing menstrual cycle regulation, promoting spontaneous ovulation, and improving pregnancy rates (81, 82). For women with obesity (BMI > 35 kg/m^2^) consideration should be given to delaying the initiation of ART treatment until weight reduction is achieved (83).

Regarding dietary modifications in conjunction with ART, a prospective study involving a diverse cohort of women undergoing IVF revealed no significant associations between carbohydrate consumption, glycemic load, glycemic index, daily fiber intake, and specific carbohydrate-rich foods intake with IVF outcomes at each procedural stage (84). On the contrary, in a prospective study on women in pre-menopause without prior infertility, the highest quintile of total carbohydrate intake and dietary glycemic load were associated with a 91% and 92% higher risk of ovulatory infertility, respectively, compared to the lowest quintile. However, intakes of fiber from various sources did not correlate with ovulatory infertility risk (85). The BioCycle study, exploring the impact of sweetened soda on estradiol concentrations in women prior to menopause, suggested that added sugars and fructose, may partially account for the negative effects of energy-containing beverages on reproductive hormones, but without affecting ovulatory function (86). The same study indicated that synthetic folate intake was linked to increased luteal progesterone levels and reduced risk of sporadic anovulatory cycles in the same study population (87).

As concerns protein intake, the consumption of plant-based proteins and the substitution of animal-derived protein with vegetable sources can boost fertility of women aged over 32 (88). However, there is no evidence to suggest that consuming dairy negatively impacts IVF outcomes; in fact, it may even be linked to increased likelihood of live birth (89). A cohort study involving women around 35 years old investigated the link between consuming protein-rich foods and outcomes of infertility treatment with ART. The results revealed that women who consumed more fish before treatment had a greater chance of live birth after an ART cycle, contributing to existing evidence correlating the intake of seafood and omega-3 fatty acids with potential enhanced fertility (90). Soy is another plant protein attracting attention for its various potential actions. While a study suggests a positive correlation between dietary soy intake and ART success in women with an average age of 35, possibly attributed to the beneficial effects of dietary isoflavones and phytoestrogens on the endometrium, more research is required to validate this hypothesis (91). The noted studies predominantly involved women of normal weight, potentially restricting the applicability of the results to other population groups.

In summary, achieving modest weight loss and incorporating specific dietary modifications, such as increasing plant-based proteins and consuming fish, may enhance fertility outcomes in women undergoing ART treatment, alongside general lifestyle improvements recommended individuals with obesity (83). Further research is needed to clarify the potential benefits of soy intake and the impact of dairy consumption on IVF outcomes.

## 6 Women with overweight/obesity and T2DM

Lifestyle interventions aimed at improving fertility in women living with obesity accompanied with T2DM are imperative given the interconnected nature of these conditions (1). Obesity precipitates a chronic low-grade inflammatory state within adipose tissue, a condition exacerbated by the expansion of adipocytes (3). This inflammatory milieu triggers IR via serine phosphorylation of insulin receptor substrate-1 (IRS-1), ultimately leading to hyperglycemia and chronic exposure to elevated blood glucose levels contributes to beta cell dysfunction (1). Moreover, the adverse effects of obesity and T2DM extend beyond metabolic disturbances, profoundly impacting reproductive health (1). Notably, women with T2DM who experience infertility often exhibit more prompt ovarian dysfunction and even encounter more challenges in achieving successful pregnancy outcomes through ART in contrast to women with infertility but without T2DM (16). It seems that insulin directly influences ovarian function, implying that IR may disrupt ovulation and endometrial structures, among others, by causing high oxidative stress, contributing to fertility challenges (1, 85). Furthermore, hyperinsulinemia is intricately linked with hyperandrogenism exacerbating endocrine dysregulation and complicating conception (1).

Given that insulin sensitivity is a pivotal factor in regulating ovulatory function and fertility in women, attention as regards nutrition should be directed towards patterns that promote insulin sensitivity and glucose metabolism to enhance fertility outcomes in women with obesity and T2DM. Irrespective of the chosen dietary regimen, achieving weight loss in this population, with diet and exercise, stands as a fundamental component in improving glycemic control and diabetes outcomes, as well as various other health metrics, such as fertility (92). This pivotal role in enhancing insulin sensitivity underscores the aforementioned mechanistic pathways linking IR, hyperglycemia, and compromised fertility.

In this direction, adherence to a MD in women of reproductive age appears promising in reducing weight gain and IR, potentially enhancing pregnancy prospects (8). Potential routes through which the MD may benefit women with IR due to T2DM and/or PCOS encompass reducing inflammatory and oxidative stress markers and enhancing the lipid profile, insulin sensitivity, endothelial function, as well as fostering anti-atherosclerotic and anti-thrombotic properties (93).

Carbohydrate aspects, particularly glycemic index and glycemic load, are central to fertility discussions, especially in the context of hyperglycemia. High-glycemic index foods and meals are implicated in glucose elevation, leading to hyperinsulinemia and IR, which are associated with higher concentrations of IGF-I and androgens (8, 24, 94, 95). This, in turn, affects ovarian function and contributes to oxidative stress, all of which detrimentally impact fertility (8, 94). Specific nutrients with a low glycemic load, such as fibers, have garnered attention for their potential impact on reproductive outcomes. Their ability to lower blood glucose levels appears to support reproduction (96). However, excessive dietary fiber intake beyond recommended levels for the general population has been linked to an elevated risk of anovulation (97). This may be attributed to reduced hormone concentrations resulting from high fiber intake, particularly the water-soluble fraction. Research suggests that for each 5 g/day increase in total fiber intake, there is a 1.78-fold increased risk of experiencing an anovulatory cycle (97). In the context of reducing carbohydrate intake, alongside focusing on quality, adopting a VLCKD has shown potential in facilitating weight loss and enhancing carbohydrate metabolism, which could potentially benefit fertility (98).

Moreover, increasing protein consumption, particularly plant-based sources over animal proteins, may restore carbohydrate-insulin balance, which, as stated above, is important in addressing anovulatory infertility in women (88, 99). The chronic consumption of a HFD in women with obesity and T2DM may enhance IR, potentially impacting reproductive function through elevated serum insulin levels constantly stimulating insulin pathways in the ovaries and pituitary (100). TFAs exacerbate inflammation and IR, thereby amplifying the risk of T2DM and metabolic disorders, including PCOS, which adversely impacts fertility (101).

In summary, a balanced, minimally processed, plant-based diet with low glycemic load meals and moderate fat and fiber intake holds promise in supporting fertility among women with obesity and T2DM, while more studies focusing on this population are necessary to comprehensively assess fertility outcomes through lifestyle modifications.

## 7 Women with overweight/obesity and hypothyroidism

Thyroid hormones are central key-players in human metabolism and the relationship between hypothyroidism and difficulty in weight loss is undisputable. However, there are several issues not well clarified in the management of woman suffering from hypothyroidism and its impact on weight loss and fertility. One major differentiation should be between subclinical (SCH) and overt hypothyroidism (OH). In the case of SCH thyroxine (T4) levels are normal and thyrotropin (TSH) values are high, whereas in OH T4 are low and TSH values elevated. Robust data have shown that human metabolism is significantly more restraint in the case of OH vs. SCH (102). However, in both cases it is advised to correct both TSH and T4 values with thyroxine administration, before the initiation of nutritional approach in order to achieve the optimal results. Beyond metabolic and weight loss alterations associated with thyroid dysfunction, elevated TRH levels aimed at boosting TSH levels, stemming from untreated thyroid disorders, can lead to conditions such as hyperprolactinemia, irregular menstrual cycles, and lack of ovulation, consequently impacting female fertility (29, 30). On the other hand, since both TSH and T4 have returned to normal levels, then the patient is considered euthyroid on thyroxine treatment. In this case, human metabolism has returned to normal and hypothyroidism is not considered as an obstacle. Nevertheless, a close follow-up at three-to-six-months intervals is advised in an attempt to obtain an ideal thyroxin substitution therapy (103).

A usual misunderstanding in hypothyroidism management is Hashimoto’s thyroiditis (HT). Autoimmune thyroiditis (Hashimoto’s) is a very common disorder affecting 5% of general population with a high prevalence in women (4:1 ratio). During its process, antithyroid autoantibodies infiltrate thyroid parenchyma leading to a progressive deterioration of thyroid function which may lead to either SCH or OH. However, in the case of a euthyroid patient with positive autoantibodies, no difference is expected on human metabolism since these autoantibodies exert their actions solely on thyroid and not in any other organ. Therefore, in these subjects no thyroxine supplementation is advised regarding the response to dietary approach (104).

The recommendation of dietary strategies offers a non-invasive avenue for achieving measurable benefits. Existing literature underscores the significant impact of diverse nutrients and dietary approaches. In individuals with obesity and thyroid dysfunction, weight reduction is deemed important due to reported associations between elevated body fat and minor alterations in TSH, free T3, and/or free T4 levels. These alterations are attributed to heightened leptin release from the adipose cells, which stimulates TSH, IR, and chronic low-grade inflammation (105). Furthermore, recent meta-analytical findings underscore a positive correlation between obesity and HT, alongside elevated levels of anti-thyroid peroxidase (anti-TPO) antibodies (106). Given that HT is not always associated with pathological values in TSH and thyroid hormones, as demonstrated in The POUNDS LOST Trial, it appears that even euthyroid patients with overweight or obesity and relatively lower levels of free T3 and free T4 may derive less benefit from dietary interventions aimed at weight loss compared to those with higher levels, and vice versa. However, thyroxine supplementation in this subgroup of patients is not advised, since well documented long term data are missing. Furthermore, treatment with thyroxine is associated with worse quality of life during treatment (107).

Recognizing the intricate interplay among obesity, thyroid function, and fertility, it becomes imperative to adopt a diet considering both quality and quantity. Notably, despite the absence of direct studies assessing the efficacy of lifestyle interventions in women with obesity and hypothyroidism concerning reproductive outcomes, the importance of such interventions remains paramount (105). A fundamental aspect of a well-balanced diet, such as the MD, involves attaining a healthy weight and restricting the intake of saturated fatty acids, sugars, and refined carbohydrates, all of which possess pro-inflammatory properties (108, 109). Essential anti-inflammatory nutrients, including vitamin D, antioxidants, monounsaturated and polyunsaturated fatty acids, magnesium, and zinc, play a significant role in mitigating thyroid inflammation (109). This concept was elucidated in a case-control study (where cases were defined as individuals with positive thyroid antibodies, irrespective of laboratory status), which revealed that dietary patterns emphasizing anti-inflammatory foods were associated with negative outcomes in plasma levels of anti-TPO and/or thyroglobulin antibodies (anti-TG). Specifically, groups consuming diets rich in vegetables, dried fruits, nuts, and muesli exhibited negative findings in anti-TPO and/or anti-TG, while those consuming diets high in animal fats and butter showed positive plasma levels of anti-TPO and/or anti-TG (p<0.05 for all findings) (110).

Additionally, iodine and selenium are crucial elements for thyroid function as they participate in the synthesis and metabolism of thyroid hormones (111). Adequate intake of iron, folic acid, and vitamin B12 is also essential due to the frequent occurrence of anaemia and cardiovascular diseases in patients with HT (109). Notably, individuals with excess weight often exhibit lower circulating levels of certain vitamins and minerals, necessitating careful attention to proper intake (105). An intervention involving a 6-month dietary regimen of 1400–1600 kcal, coupled with daily supplementation of selenium and zinc (2–4 times higher than the Recommended Dietary Allowance for the general population), in women with obesity previously diagnosed with HT and receiving levothyroxine treatment (with one-third on euthyroid status at baseline), resulted in significant reductions in weight, BMI, body fat percentage, as well as TSH, anti-TG, and anti-TPO levels, alongside a concurrent increase in free triiodothyronine (fT3) and free thyroxine (fT4) levels (112).

According to current knowledge, the elimination of gluten or lactose is recommended in cases of food intolerances or diseases such as celiac disease (113). Notably, individuals requiring high doses of levothyroxine or experiencing resistance to treatment, coupled with challenges in regulating TSH levels, should be considered for lactose or gluten intolerance testing (114). Both lactose and gluten intolerance are associated with damage to intestinal villi, leading to an increased demand for levothyroxine (114). Moreover, patients with HT are predisposed to immune-related conditions like gluten sensitivity, thus prompting consideration of an elimination diet if screening for such conditions yields positive results (109).

Krysiak et al. explored the effects of a 6-month gluten-free diet on euthyroid untreated patients with HT and positive anti-tissue transglutaminase antibodies (anti-tTG), albeit without clinical symptoms or diagnosed celiac disease. The study found that the gluten-free diet significantly decreased levels of anti-TPO and anti-TG antibodies in the experimental group compared to the control group (without a gluten-free diet). This enhancement in antibody titers may be attributed to observed improvements in vitamin D levels, as suggested by the authors (115). Intriguingly, another study aimed to evaluate the effectiveness of a gluten-free diet in patients with chronic autoimmune thyroid disease without positive celiac antibodies or gluten intolerance. This study revealed that the gluten-free diet led to reductions in TSH levels and increases in fT4 levels compared to the control group, suggesting a potential improvement in intestinal absorption of levothyroxine (116). Additionally, logarithmic transformation analysis demonstrated improvements in anti-TG, TSH, and fT4 levels at 3, 6, and 12 months post-intervention, as well as in anti-TPO only in the third month of the trial. Consequently, the authors proposed that after excluding celiac disease, a gluten-free diet may be considered, although clear evidence supporting its recommendation for every patient with HD or other autoimmune diseases with shared pathophysiological pathways remains lacking, necessitating further research to evaluate its efficacy (116).

Regarding lactose, its elimination from the diet appears beneficial in reducing TSH levels in lactose-intolerant patients with HT receiving levothyroxine treatment, but not in lactose-tolerant individuals (114). However, other exclusion diets, such as the avoidance of goitrogenic foods or strict limitation of carbohydrates, lack substantial data and should not be considered as primary interventions, both in patients with eu- and hypo- thyroidism.

Overall, optimizing thyroid hormone levels alongside dietary modifications rich in anti-inflammatory nutrients such as vitamin D, antioxidants, monounsaturated and polyunsaturated fatty acids, magnesium, and zinc and limited to specific food elements only when there is a medical reason is essential for addressing metabolic health, weight loss, and fertility concerns in women with hypothyroidism, particularly those with HT.

## 8 Eating disorders

EDs are prevalent among women of reproductive age, typically emerging before the age of 25 (117). This onset often predates pregnancy attempts, leading to a well-documented association between EDs and reduced fertility (118). It has been demonstrated that a significant proportion of women seeking fertility assistance exhibit ED-related issues (119, 120), and those with a history of EDs commonly report difficulties conceiving (121). Studies have linked ED psychopathology with long-term fertility concerns, particularly in individuals previously diagnosed with anorexia nervosa (AN) or bulimia nervosa (BN) or eating disorder not otherwise specified (EDNOS), who demonstrate delayed first births and lower parity compared to those with absence of EDs (122).

The adverse effects of EDs on reproductive health stem from associated physical consequences such as extreme body weight fluctuations and menstrual irregularities (118, 123, 124). While menstrual disturbances are not exclusive to a specific type of ED (125), prolonged disruptions are predominantly observed in individuals with AN (126). However, women with binge-eating disorder (BED) also experience these disruptions (127). Additionally, both AN and BED can lead to ovulation suppression in severely affected individuals, contributing to approximately 60% of cases of anovulatory infertility (122). Disturbances in the hypothalamus-pituitary-gonadal axis, resulting from irregular eating behaviors and undernutrition, further exacerbate infertility in individuals with EDs (128). Leptin resistance or tolerance existing in cases of excess weight may partially contribute to this, impeding its function in addressing abnormalities in the hypothalamic-pituitary-peripheral axes, involving the gonadal, thyroid, growth hormone, and to a lesser extent adrenal axes (129).

Regarding the interplay between obesity and EDs, BED and BN have been the primary focus of research in individuals with obesity (130). Women with a background of BN demonstrate a higher likelihood of childlessness compared to their counterparts and experience elevated rates of miscarriage in comparison to women with AN (131, 132).

Cousins et al. observed heightened drive for thinness and bulimic symptoms among women seeking treatment for ovulatory and unexplained infertility compared to non-infertile women receiving primary care, suggesting that premorbid ED symptoms might contribute to nutritional deficits and hormonal irregularities predisposing them to fertility difficulties (120). An observational study employing a Nutrition Screening Form (NSF) aimed at identifying lifestyle risk factors detrimental to fertility uncovered that 43% of the participants had a BMI <20 or ≥25 kg/m^2^, both recognized as infertility risks. Additionally, nearly half reported engaging in dieting and having unrealistic weight goals, potentially compromising energy and essential nutrient intake. A significant proportion disclosed a history of EDs, vegetarianism, low-fat or low-cholesterol diets, and dietary supplement use. Moreover, 14% lacked folic acid supplementation, 13% reported extremely or very active exercise levels, and 28% reported high levels of perceived stress. Thus, modifying these lifestyle factors, many of which may be associated with (sub)clinical EDs, is imperative, following heightened awareness among healthcare professionals and patients regarding nutrition and exercise behavior and successful reproductive outcomes (133).

Clinicians should take into account the type of ED and family fertility histories when addressing the long-term reproductive health needs of women previously diagnosed with AN, BN, or EDNOS (122). Whenever feasible, diagnosing and treating EDs before conception is advisable, with a caution against utilizing fertilization treatment in patients who refuse psychological treatment (22). Recognizing and referring these women for ED treatment can mitigate the medical and psychiatric consequences of EDs on both the woman and her offspring. If infertility appears linked to ED behavior, prioritizing referral to ED-specific psychological treatment by a specialist is crucial, potentially averting unnecessary fertility interventions (134). Particularly in patients with EDs and underweight, ART is contraindicated, highlighting the importance of reducing over-exercising and improving nutrition and body weight (135). Key components for management of this group include ED support, ensuring adequate nutrition and hydration, guidance on healthy eating patterns, and strategies to address specific ED behaviors (134). A multidisciplinary approach involving mental health care, registered dietitians, and specialized medical professionals is essential (134, 135). However, there are currently no specific clinical recommendations available for gynecologists or fertility specialists regarding the management of females seeking fertility treatment or pregnant females with EDs (135).

Dietitians specialized on these cases should prioritize achieving weight restoration and addressing nutrient deficiencies, while preventing or managing refeeding syndrome and its potential complications in women with AN (136). The immediate treatment goals for all patients with EDs include interrupting compensatory behaviors. Subsequently, the aim shifts towards restoring meal patterns promoting social interaction, broadening the food repertoire, and achieving a balanced macronutrient intake (136). Additionally, it is considered essential to assist patients in rediscovering a healthy relationship with food and to overcome fears surrounding eating (136). Importantly, weight restoration alone is insufficient for achieving complete recovery; addressing distorted body image, ED thoughts/behaviors, psychological and psychiatric comorbidities, and social or functional impairments is equally vital during ED treatment (136). Appropriate ED treatment and weight restoration appear to mitigate the impact of EDs on reproductive health (137).

## 9 Conclusion

In conclusion, addressing obesity through lifestyle modifications, including regular exercise and balanced nutrition, holds promise in improving fertility outcomes in women with excess weight, particularly those with associated conditions such as PCOS, CAH, T2DM, premenopause, hypothyroidism and EDs. While maintaining a healthy weight and adhering to general lifestyle recommendations are necessary, individualized approaches considering concurrent conditions, personal needs, preferences, and cultural backgrounds are essential. Lifestyle interventions, due to their cost-effectiveness and potential benefits, should be prioritized. Although robust data linking specific endocrine problems with positive lifestyle changes to improve fertility may be lacking, it is prudent to integrate existing findings and emphasize relevant principles in clinical practice. By promoting comprehensive lifestyle changes, healthcare providers can play a vital role in managing fertility issues in women with obesity and associated conditions, ultimately leading to improved reproductive outcomes and overall well-being.

## Author contributions

EG: Writing – review & editing, Writing – original draft, Visualization. SL: Writing – review & editing, Writing – original draft. GA: Writing – review & editing.
